# Nursing at St. Mary's Hospital

**Published:** 1886-11-06

**Authors:** P. Michelli

**Affiliations:** The Secretary


### A Noble Profession: Nursing the Sick.

### NURSING AT ST. MARY'S HOSPITAL.

By the Secretary, Mr. P. Michelli.

Introductory. The system of nursing, if system it can be called, that was in vogue during the early part of the present century in the London hospitals was primitive in the extreme. No special training seems to have been considered necessary until Miss Florence Nightingale drew the attention of the civilised world to the subject after the Crimean war. Not much improvement had taken place in 1851, when this hospital was first opened; it seemed to be considered then that anyone could act as a nurse. In one particular, and in that one alone, I believe, some slight improvement had been made, and that was the engagement of younger women to tend the sick. These were more likely to learn and profit by the few hints thrown out by physician or surgeon; but there it ended, and until 1856 the subject seems to have hardly attracted the attention of anyone. Hygienic surroundings were but little understood, cleanliness was a secondary consideration, and the patients got well in the intervals of the doctor's visits as best they could, or else succumbed. When this hospital first opened its wards, no system of training nurses was in existence. The best attendants that could be had were obtained, and well they seem to have done their work, considering the disadvantages under which they laboured. A few had some little experience in Poor-law Infirmaries, but the rest were engaged as "raw hands," with no conception of the work required of them.

The First Probationer in 1861. The first probationer, as we understand the word, *i.e.*, one who comes simply to learn under the supervision of an experienced teacher, was a Miss Rokeby, who entered the hospital in 1861. Mrs. Wright was at this time matron, a lady of some ten years' experience, and Miss Rokeby appears to have remained at the hospital under instruction for twelve months. Probationers from this time were received at the hospital for periods varying from three to twelve months; no certificates were granted, but the matron gave a written testimonial. Each received board and lodging, without uniform or wages, and about sixty nurses were so trained.

Nurses Trained for other Institutions. In September 1867 the London Training School and Home for Nurses on Clapham Common (afterwards the British Nursing Association, of 4, Trafalgar Square, W.C., and Cambridge Place, W., which ceased to exist in 1883), of which Sir Arthur Laurence, K.C.B., was chairman, requested the governors to allow one or more probationers to be trained for them at St. Mary's. The governors consented, providing a fee of £26 per annum was paid for each probationer to defray the cost of her board, and that the number should not exceed six at one time. This arrangement lasted until January 1870, with slight modifications, when the agreement was terminated. The governors also entered into a similar agreement in 1876 with the Norfolk and Norwich Staff of Hospital Trained Nurses. There has generally been an average of four probationers always under training in the hospital for this Institution. Nurses have also been trained for the Leamington Infirmary, the Worcester Nurses' Home, and the Metropolitan and National Nursing Association.

A New Departure. In 1874 a new code of rules and regulations for improving the nursing was introduced by Mrs. Wemyss Anderson, a lady much interested in the hospital. This was approved by the weekly board, and much greater accommodation was then afforded. The dormitories were enlarged, the sitting-room improved, and an extensive addition made to the dining hall. The system now in vogue was then adopted, that probationers should receive a small annual payment, together with board and lodging during their training, on the condition that they enter the service of the hospital afterwards as regular nurses, a certificate at the end of the second year being granted. Subsequently lady probationers were received. They pay a fee of £30, and receive their certificate at the expiration of their year's training, and can then leave the hospital. On a further payment of £20 a separate bedroom is provided. Miss Williams, who was matron from 1876 to 1884, did much to improve the nursing. "Staff-nurses" were for the first time substituted for "first-class nurses," and wardmaids were engaged to do the rough work of the wards, which had hitherto been performed by the regular nurses. Courses of lectures on medical and surgical nursing are given by members of the staff to the nurses, and examinations in the subjects treated of are held at the expiration of the courses. Ladies unconnected with the hospital are allowed to attend these lectures on payment of a small fee.

Honours and Promotion. The majority of the sisters and nurses at present in the wards have been trained in the hospital. One of the probationers, Miss Norman, served through the first Egyptian campaign, and received at the hands of Her Majesty the Royal Red Cross. St. Mary's has besides numbered among its probationers many who have done equal credit to the school at which they were trained. Of these may be mentioned Mrs. Wemyss Anderson, Hon. Katherine Scott, Miss Power, Lady Amberley, Miss McLaughlin, Baroness Ebba Bostron, Miss Byam, Miss

Hind, Miss Wrigley, Lady Katherine Gordon, Miss Machin, Miss Dowse, and Miss Kerr. Many now hold high appointments in the hospital world, others have served with credit through campaigns, and all have done us credit. It will be seen that St. Mary's was one of the pioneers in the nursing world, and now, under the management of Miss Medill, the present matron, the nursing at the hospital appears to be more popular than ever. This is evinced by the daily increasing number of applications which are received from ladies wishing to be trained as nurses.

